# Effects of nitrate- and ammonium- nitrogen on anatomical and physiological responses of *Catalpa bungei* under full and partial root-zone drought

**DOI:** 10.1186/s12870-024-04874-3

**Published:** 2024-03-26

**Authors:** Ting Xu, Zhiyong Wang, Ziye Wang, Mengfan Guo, Xintong Wang, Xuelian He, Junhui Wang, Siddiq Ur Rahman, Mohammed Bourhia, Abdulaziz Abdullah Alsahli, Yi Zhang

**Affiliations:** 1 State Key Laboratory of Crop Stress Biology in Arid Areas, Northwest A&F University, Yangling, Shaanxi 712100 China; 2 State Key Laboratory of Tree Genetics and Breeding, Chinese Academy of Forestry, Beijing, 100091 China; 3 Department of Computer Science and Bioinformatics, Khushal Khan Khattak University, Karak, Khyber Pakhtunkhwa 27200 Pakistan; 4 Laboratory of Biotechnology and Natural Resources Valorization , Faculty of Sciences, Ibn Zohr University, Agadir, 80000 Morocco; 5 Department of Botany and Microbiology, College of Science, King Saud University, Riyadh, 11451 Saudi Arabia

**Keywords:** Heterogeneous drought, Ammonium, Nitrate, Antioxidant defense, *WUE*_i_, *CATALPA bungei*

## Abstract

*Catalpa bungei* is a precious timber species distributed in North China where drought often occurs. To clarify adaptive responses of *C. bungei* to partial- and full- root-zone drought under the influence of nitrogen forms, a two-factor experiment was conducted in which well-watered (WW), partial root-zone drought in horizontal direction (H-PRD) and in vertical direction (V-PRD), and full root-zone drought (FRD) were combined with nitrate-nitrogen (NN) and ammonium-nitrogen (AN) treatments. *C. bungei* responded to FRD by sharply closing stomata, decreasing gas exchange rate and increasing leaf instantaneous water use efficiency (*WUE*_i_). Under FRD condition, the growth of seedlings was severely inhibited and the effect of N forms was covered up by the drastic drought effect. In comparison, stomata conductance and gas exchanges were moderately inhibited by PRDs. *WUE*_i_ in V-PRD treatment was superior to H-PRD due to the active stomata regulation resulting from a higher ABA level and active transcription of genes in abscisic acid (ABA) signaling pathway under V-PRD. Under both PRDs and FRD, nitrate benefited antioxidant defense, stomata regulation and leaf *WUE*_i_. Under V-PRD, *WUE*_i_ in nitrate treatment was superior to that in ammonium treatment due to active stomata regulation by signaling network of nitric oxide (NO), Ca^2+^ and ABA. Under FRD, *WUE*_i_ was higher in nitrate treatment due to the favoring photosynthetic efficiency resulting from active NO signal and antioxidant defense. The interactive effect of water and N forms was significant on wood xylem development. Superoxide dismutase (SOD) and catalase (CAT) largely contributes to stress tolerance and xylem development.

**Supplementary Information:**

The online version contains supplementary material available at 10.1186/s12870-024-04874-3.

## Introduction

*Catalpa bungei* is a genus of the family *Bignoniaceae* and it is an excellent native tree with a large ecological range in northern part of China [1, 2]. *C. bungei* is a high economic timber species characterized by fast growth rate, powerful bending resistance [3, 4] and good quality wood. The wood of *C. bungei* has great durability and corrosion resistance which is often utilized to make furniture, boats, and other high-grade wood products in ancient China. *C. bungei* is mainly distributed in North China and Central China with annual rainfall of 400–1000 mm and frequent drought. Although production management methods such as pruning lateral branches of young plantations and controlling planting density below 500 plants per hectare can increase the wood yield of *C. bungei* [5], water is the major environmental factor that limits wood production of *C. bungei* [6, 7]. It is of great importance to disclose how *C. bungei* cope with and acclimate to water deficit and maintain wood productivity.

To cope with drought stress, woody plants usually exhibit altered anatomical features, activation of ABA signaling, up-regulation of plasmam embrane intrinsic proteins (*PIPs*) transcription and increased production of osmotic adjustment substances such as soluble sugars, amino acids, and free proline and antioxidants including SOD, guaiacol peroxidase (GPX) and ascorbate peroxidase (APX) [6, 8, 9]. The study on poplar found that the transcript levels of *PIP2;3*, *PIP2;4*, 9-cis-epoxycarotenoid dioxygenase (*NCED3*) and protein phosphatase 2 C (*PP2C*) were induced under severe drought in roots of *Populus* × *euramericana* and *Populus cathayana*, and the thickness of foliar spongy tissue was increased under drought in both genotypes [8]. Most of the previous studies about drought tolerance of tree species were conducted under the premise that soil water was evenly distributed across the soil profile and thus drought occurs in a homogeneous pattern. However, soil moisture distribution in soil is usually heterogeneous due to precipitation, evaporation and human activity such as irrigation. Therefore, a part of plant root is usually located in moist soil zone while the other part is in dry zone [10, 11]. Irrigation water has become increasingly scarce in many areas resulting from ever-increasing scale of agricultural and industrial production. In agriculture and horticulture soil, a water-saving irrigation technology named alternate partial root-zone irrigation (PRI) produces PRD [12, 13]. In PRI, drought stress is perceived by the roots in the drought soil zone, which may induce the production and root-shoot transport of drought signals such as ABA and leading to partial closure of stomata in leaves, so as to reducing the water losses via leaf transpiration [13–15]. At the same time, root system in the region of wet-soil can acquire considerable amount of water to afford plants growth and development [16–18].

As compared with agricultural soil, the spatial distribution of water in forest soil was far more complex and PRD may occur frequently in both horizontal direction (H-PRD) and vertical direction(V-PRD) [11]. In forest soil with limited irrigation and tillage, surface soil usually dry faster than deeper soils due to higher evaporation rate and faster water consumption rate by plants. As a result, heterogenous soil moisture distribution in vertical orientation is particularly common [19]. Given the universality and complexity of water heterogeneity in forest soils, it is particularly important to elucidate how woody plants acclimate to partial root-zone drought in both horizontal- and vertical- directions.

Nutrient drives changes in functional traits of plants and thus impact the degree of drought tolerance [20]. Nitrogen (N) is closely associated with the development of drought tolerance functions including net photosynthetic rates (*A*), intrinsic water use efficiency (*WUE*_i_), hydraulic conductance, and antioxidant defenses [21–24]. Nitrate (NO_3_^−^) and ammonium (NH_4_^+^) are the two dominant inorganic nitrogen forms in soil, which have different effects on plant morphological, physiological and biochemical processes. Many plant species have different preferences for NH_4_^+^ or NO_3_^−^ [25, 26]. It was reported that the biomass of *Solanum melongena* under partial root-zone irrigation were significantly increased by nitrate treatment [27]. Our previous study has elucidated how N availability (deficient-N or sufficient-N) affects drought response of *C. bungei* under differential root-zone drought [28]. However, drought physiology of *C. bungei* under the influence of different N forms (nitrate or ammonium) is still unknown.

Here, a green-house experiment was conducted in which partial root-zone irrigation was designed and split-root pots were used. The objective was to address the following hypotheses: (1) *C. bungei* acclimate to full and partial root-zone drought by specific mechanisms at physiological and anatomical levels and (2) different N forms (nitrate or ammonium) influence drought responses of *C. bungei* under full and partial root-zone drought.

## Materials and methods

### Plant material and experimental design

The experimental material of *Catalpa bungei* (clone ID, “2”) was cultured in the greenhouse of Northwest A & F University, Yangling, China (34°20′N, 108°24′E). The rooted seedlings of 10 cm height in the tissue culture bottle were transferred to the climate chamber for incubation. The humidity of the climate chamber was set at 50–60%, with a daytime temperature of 26 °C and a nighttime temperature of 20 °C and 14 h of light per day. After three days of culture, the rooted seedlings were transplanted to split-root pots (10 L) with mixed substrates (sand: soil, 1:2, v/v) in a glasshouse under the same surroundings as the climatic chamber. The soil in the mixed substrate was a nutrient-depleted loess with low levels of hydrolyzed nitrogen (21.2 mg kg^− 1^), available phosphorus (3.55 mg kg^− 1^) and potassium (12.3 mg kg^− 1^), and the pH of the mixed substrate was 7.8.

A two-factor design was used, in which four water treatments were combined with two N conditions. The four water conditions were WW (well-watered), H-PRD (partial-root horizontal region drought), V-PRD (partial-root vertical region drought) and FRD (full root- region drought). The two nitrogen conditions were NN (nitrate-N) and AN (ammonium-N). The experiment was set up with 8 treatments, and a total of 32 seedlings were included in this experiment (8 treatments × 4 replicates). After 20 days of preculture with modified Long Ashton (LA) nutrient solution (0.9 mM CaCl_2_·2H_2_O, 0.5 mM KCl, 1.0 mM NH_4_NO_3_, 0.3 mM MgSO_4_·7H_2_O, 0.6 mM KH_2_PO_4_, 0.042 mM K_2_HPO_4_, 2µM MnSO_4_·4H_2_O, 10µM H_3_BO_3_, 7µM Na_2_MoO_4_, 0.05µM CoSO_4_, 0.2µM ZnSO_4_, 0.2µM CuSO_4_, 0.01mM Fe-EDTA), the seedlings were treated with nitrate-N or ammonium-N, in which N resources in LA solution was replaced by 2.0 mM Ca(NO_3_)_2_ (nitrate-N) or 4.0 mM NH_4_HCO_3_ (ammonium-N), respectively. The application of LA to each pot was 100 ml per pot every 3 days. The seedlings were treated with nitrogen for 90 days.

Water treatment was applied after 60 days of nitrogen treatment and it was lasted for 30 days. The soil moisture content of WW treatment was 70 ± 5% of field capacity. In H-PRD treatment, soil in each pot was divided into two equal halves, i.e. drought root zone and moist root zone, by placing a plastic board in the middle of the pot (Fig. S1). The soil moisture content of drought root-zone and moist root-zone in H-PRD treatment was set to 45 ± 5% and 70 ± 5% of field capacity, respectively (Fig. S1). In V-PRD treatment, soil in each pot was divided into upper-half and lower half, in which the soil moisture content was set to 45 ± 5% and 70 ± 5% of field capacity, respectively (Fig. S1). To achieve the soil moisture described above, drip irrigation device with water dropper in each compartment was applied. One water dropper was set in the drought root zone and moist root zone of the H-PRD treatment, respectively. Similarly, one water dropper was set in the upper-half and lower-half of soil in the V-PRD treatment, respectively (Fig. S1). The soil moisture content of FRD treatment was 45 ± 5% of field capacity on the whole soil region and it is classified as severe drought stress according to the classification of plant water gradient proposed by Hsiao [29]. The time domain reflectometry apparatus (TDR300) was applied to detect soil water contents in different soil zones every day. The water flow in each water dropper was set to a slow rate to avoid water diffusion between different root zones.

### Measurement of growth and gas exchanges

At the end of all experimental treatments, the major growth parameters including basal diameter and tree height were measured using a tape measure and vernier caliper. The R/S was the ratio of root to shoot DW. Gas exchange parameters such as transpiration rates (*E*), net photosynthetic rates (*A*), stomatal conductivity (*g*_s_) and intercellular carbon dioxide concentration (*CO*_2int_). were measured on three mature and healthy leaves from each seedling using the portable photosynthesis equipment (Li-6400, Licor Biosciences, Lincoln, NE, USA). The measurements were taken from 09:00 to 11:00 h. The *WUE*_i_ was calculated as *A* divided by *E*. The intrinsic water use efficiency (*iWUE*) was the ratio of *A* to *g*_s_. Subsequently, the samples were quick-frozen by liquid N and then placed in a refrigerator (-80 °C) for the subsequent analysis at physiological and transcriptional levels. Dry weights of leaves, stems and roots were measured after drying the tissues at 70 °C for 72 h. Subsequently, each organ biomass of each sapling was measured. Relative water content in the leaves was determined based on the method described by reported research [30].

### Measurement of phytohormones and antioxidants

Each leaf sample was grinded into powder using liquid nitrogen. An ultra performance liquid chromatography-tandem mass spectrometry (UPLC-MS/MS) method was used to measure concentration of phytohormones including salicylic acid (SA), ABA, indole acetic acid (IAA), and gibberellic acid (GA_3_). The level of soluble protein in the leaf samples was measured using the method reported by Bermejo et al. [31]. The content of glutathione (GSH) was measured by 5,5 ‘- Dithio bis- (2-nitrobenzoic acid) (DTNB) - GR recovery process [32]. In the determination of superoxide dismutase (SOD) activity (EC 1.15.1.1), a unit of SOD was determined as the enzymatic quantity that reduced the SOD-inhibited nitroblue tetrazolium reductase by 50% at 550 nm [8]. The concentration of free proline was assayed with spectrophotometric methods following the description of Shi et al. [6]. The activities of the APX and CAT were measured [33].

### Measurement of NO and Ca^2+^ signals

The concentration on NO in leaves was assayed by Griess reagent following a method provided by Zhang et al. [34]. Absorbency was determined with a spectrophotometer (540 nm). Through matching with the specification curve based on NaNO_2_ as standard, the amount of NO was assayed. The concentration of Ca^2+^ (mg⋅g^− 1^) was assayed with a calcium colorimetric assay kit (Beyotime Biotechnology Co, Ltd. Shanghai, China).

### Anatomical analysis of stem xylem

When the samples were harvested whole, stem segments located at 20% distance from stem apex to stem base were selected and cut into 2–3 mm cylindrical pieces and fixed with FAA fixation fluid (70% ethanol: glacial acetic acid: formalin, 90:5:5). The paraffin sections were prepared according to the steps of dehydration, transparency, wax immersion, sectioning (10 μm) and staining. The cross section of the stem was observed by optical microscopy and the finding was photographed and noted. Microscopic image processing software (Image J, National Institutes of Health, USA) was utilized for measuring xylem width, number of xylem cell layers, diameter of xylem cells, diameter of vessel and phloem width.

### Transcriptional analysis of genes participating in drought response

Transcriptional abundance of genes in ABA signaling pathway such as *NCED*, ABA-8′-hydroxylase, *PYL2*, *ABAI-5* (ABA insensitive-5) related to drought tolerance was measured by qPCR. and *PIP* genes including *PIP1-3*, *PIP2-3*, *PIP2-4*, *PIP2-5* and *PIP2-7* were selected for qPCR analysis as they were essential in regulation of water flow across the plasmalemma in plant [35]. The RNA isolation of leaves was used by plant RNA extraction kits (Takara, Japan) and converted into cDNA by utilizing the cDNA reverse transcription kit (Takara, Japan). Quantitative PCR was used in a 20 µl reaction volume, which contained 0.5µM of each primer, 2.5 µl cDNA and 10 µl 2× SYBR Green Premix EX Taq II (Takara). PCR amplification was performed by IQ5 Real-Time PCR machine (Bio-Rad, Hercules, CA, USA). Four independent biological replicates from each treatment were selected for qPCR analysis, and each sample consisted of three technical replicates. The *β-tubulin* was selected as reference gene. The transcription level of the genes was expressed by calculating the difference between the Cq value of the reference gene and the Cq value of the target gene. Visualization of gene expression was performed by the software of HemI (Heatmap Illustrator).

### Statistical analysis

The two-way ANOVAs of SAS software (SAS Institute, Cary, NC) was used to indicate the effect of water and nitrogen treatments on the test variables. Experimental data were tested using the UNIVARIATE procedure for normality before statistical analysis. Differences among treatments were determined by using multiple mean comparisons (Fisher’s LSD test). *P* < 0.05 was defined as significant difference (ANOVA F-test). Pearson’s correlation and principal component analysis were performed by using R.

## Results

### Seedling growth, water status and gas exchange parameters under partial root-zone drought as effected by N forms

**Fig. 1:**
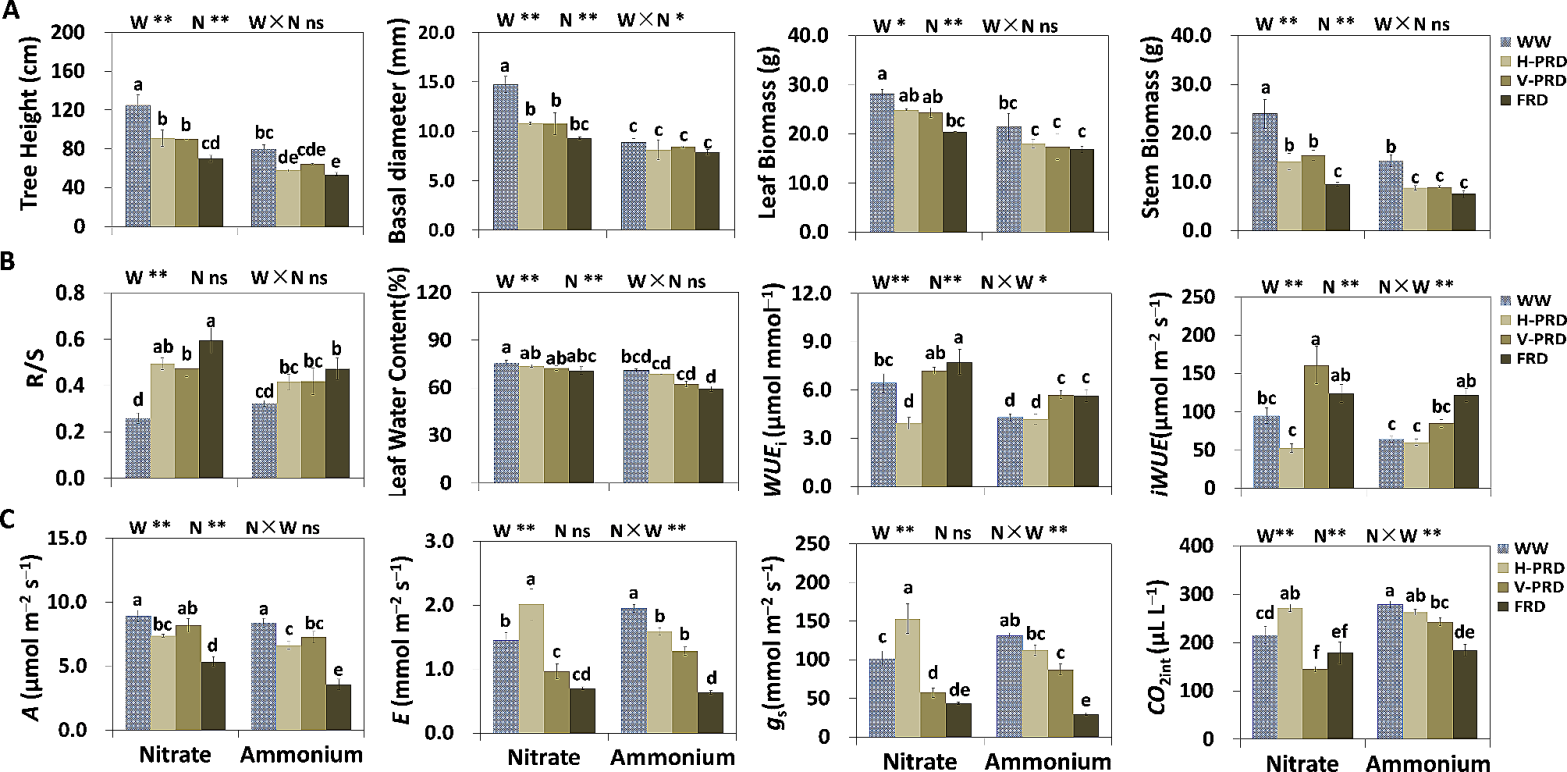
Effects of partial root-zone drought and different N forms on growth, water status and gas exchange parameters of seedlings. (**A**) Statistical analysis of growth traits. (**B**) Statistical analysis of plant water status. (**C**) Statistical analysis of gas exchange parameters. The four water conditions (well-watered condition (WW), partial-root zone drought in horizontal (H-PRD) and vertical (V-PRD) direction, and full root zone drought (FRD)) are combined with nitrate (NN) or ammonium (AN) treatment. Means ± SE are indicated by the columns and the bars above columns (*n* = 4). Statistically different values (*P* < 0.05) based on the multiple comparisons (Fisher’s LSD test) in ANOVA are indicated by different letters above the bars. The results of two-way ANOVA (water effect, nitrogen effect, and interaction effect) are listed as: *P < 0.05; **P < 0.01; ns, not significant

In nitrate treatment, stem biomass, tree height and basal diameter were inhibited by all of the three drought conditions (Fig. 1A). Leaf biomass was decreased by FRD but not by PRDs (Fig. 1A). FRD reduced the leaf biomass 28% compared to control in nitrate treatment and 22% in ammonium treatment. The leaf biomass, tree height and R/S were higher (20%, 31% and 25%, respectively) in nitrate treatment compared to ammonium under FRD (Fig. 1A, B). Stem biomass and seedling height both were decreased by H-PRD and FRD, and stem biomass was decreased by V-PRD in ammonium (Fig. 1A). For leaf water content, it was higher it was higher (7%, 8%, 16% and 20%, respectively) in nitrate treatment compared to ammonium under all water conditions (Fig. 1A). N forms did not affect basal diameter and biomass of stem and leaf under FRD (Fig. 1A). All growth traits in nitrate treatment were superior to ammonium under WW and PRDs conditions (Fig. 1A).

Gas exchange parameters largely decreased upon FRD in both N forms (Fig. 1C). *A* was remarkably decreased by all drought conditions in both N treatments, and the degree of inhibition was greater under FRD treatment (Fig. 1C). Under FRD, *A* was decreased by 40% in nitrate treatment and 46% in ammonium treatment, and *A* was higher 50% in nitrate treatment compared to ammonium (Fig. 1C). *WUE*_i_ was higher (26% and 37%, respectively) in nitrate treatment compared to ammonium under V-PRD and FRD (Fig. 1B). *E*, *g*_s_ and *CO*_2int_ were higher in ammonium treatment under WW and V-PRD (Fig. 1B, C), and *E*, *g*_s_ and *iWUE* were higher in nitrate treatment under H-PRD treatment (Fig. 1B, C).

### Antioxidants and signals of NO and Ca^2+^ as affected by nitrogen forms

**Fig. 2:**
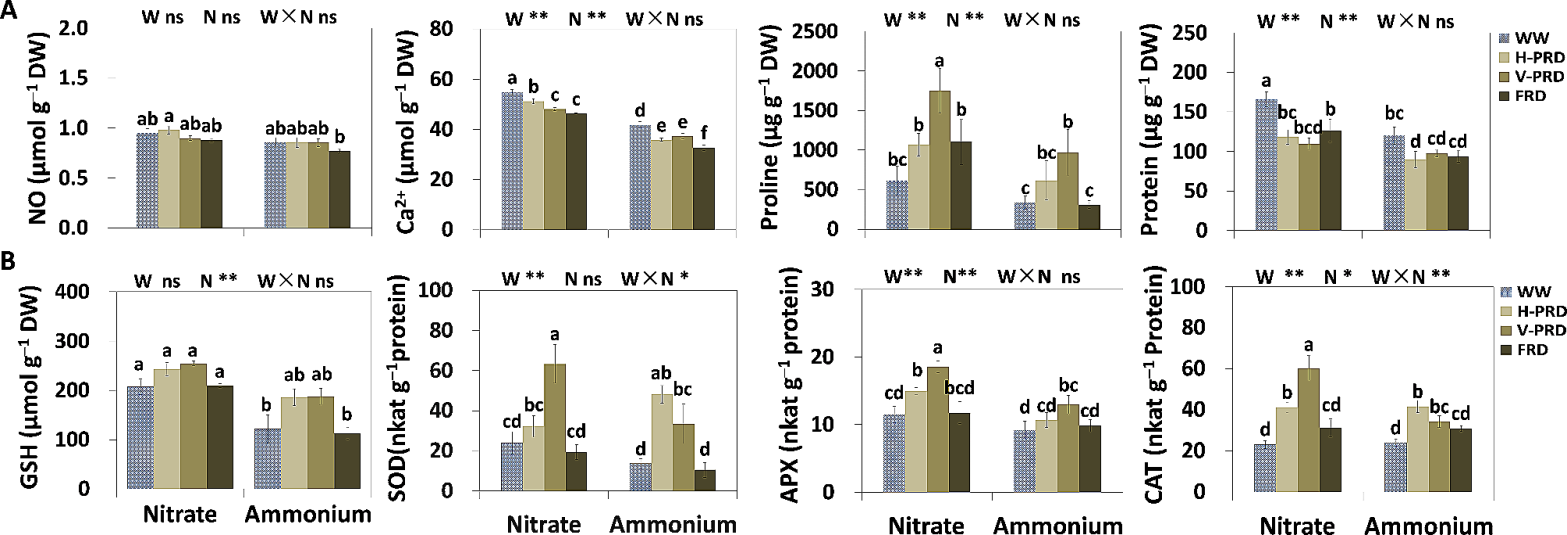
Relevant signals and defense mechanisms induced by drought. (**A**) Content of free proline, soluble protein, NO and Ca^2+^ in leaves. (**B**) Content of GSH, soluble protein, SOD, APX and CAT activity in leaves under the combined conditions of water and N forms. The four water conditions (well-watered condition (WW), partial-root zone drought in horizontal (H-PRD) and vertical (V-PRD) direction, and full root zone drought (FRD) are combined with nitrate (NN) or ammonium (AN) treatment. Means ± SE are indicated by the columns and the bars above columns (*n* = 4). Statistically different values (*P* < 0.05) based on the multiple comparisons (Fisher’s LSD test) in ANOVA are indicated by different letters above the bars. The results of two-way ANOVA (water effect, nitrogen effect, and interaction effect) are listed as: *P < 0.05; **P < 0.01; ns, not significant

There was significant effect of nitrogen forms on the concentration of Ca^2+^, free proline, soluble protein, and GSH and on the activity of APX and CAT (Fig. 2). Under both PRDs and FRD, the level of NO was higher (15%, 5% and 14%, respectively) in nitrate than that in ammonium treatment (Fig. 2A). The level of Ca^2+^ was higher (31%, 43%, 29% and 42%, respectively) in nitrate than that in ammonium treatment under all water conditions, and Ca^2+^ concentration was inhibited by PRDs and FRD (16% and 22%, respectively) in both N treatments (Fig. 2A). Significant effect of water treatment was found for the activity of SOD, APX and CAT and on the level of soluble protein and free proline (Fig. 2A, B). The level of free proline, APX and CAT were increased by all drought treatments regardless of N forms (Fig. 2A, B). Under WW treatment, GSH and soluble protein were higher (70% and 39%, respectively) in nitrate than that in ammonium treatment in nitrate (Fig. 2A, B). Under V-PRD, the level of free proline, SOD, APX and CAT were higher (80%, 90%, 38% and 76%, respectively) in nitrate compared to ammonium treatment (Fig. 2A, B). Under H-PRD treatment, soluble protein and APX in nitrate treatment were higher (72% and 41%, respectively) (Fig. 2A, B). Under FRD, the activity of GSH and soluble protein were higher (84% and 34%, respectively) in nitrate than that in ammonium treatment (Fig. 2A, B).

### Phytohormones affected by nitrogen forms and transcription level of critical genes related to drought physiology

**Fig. 3:**
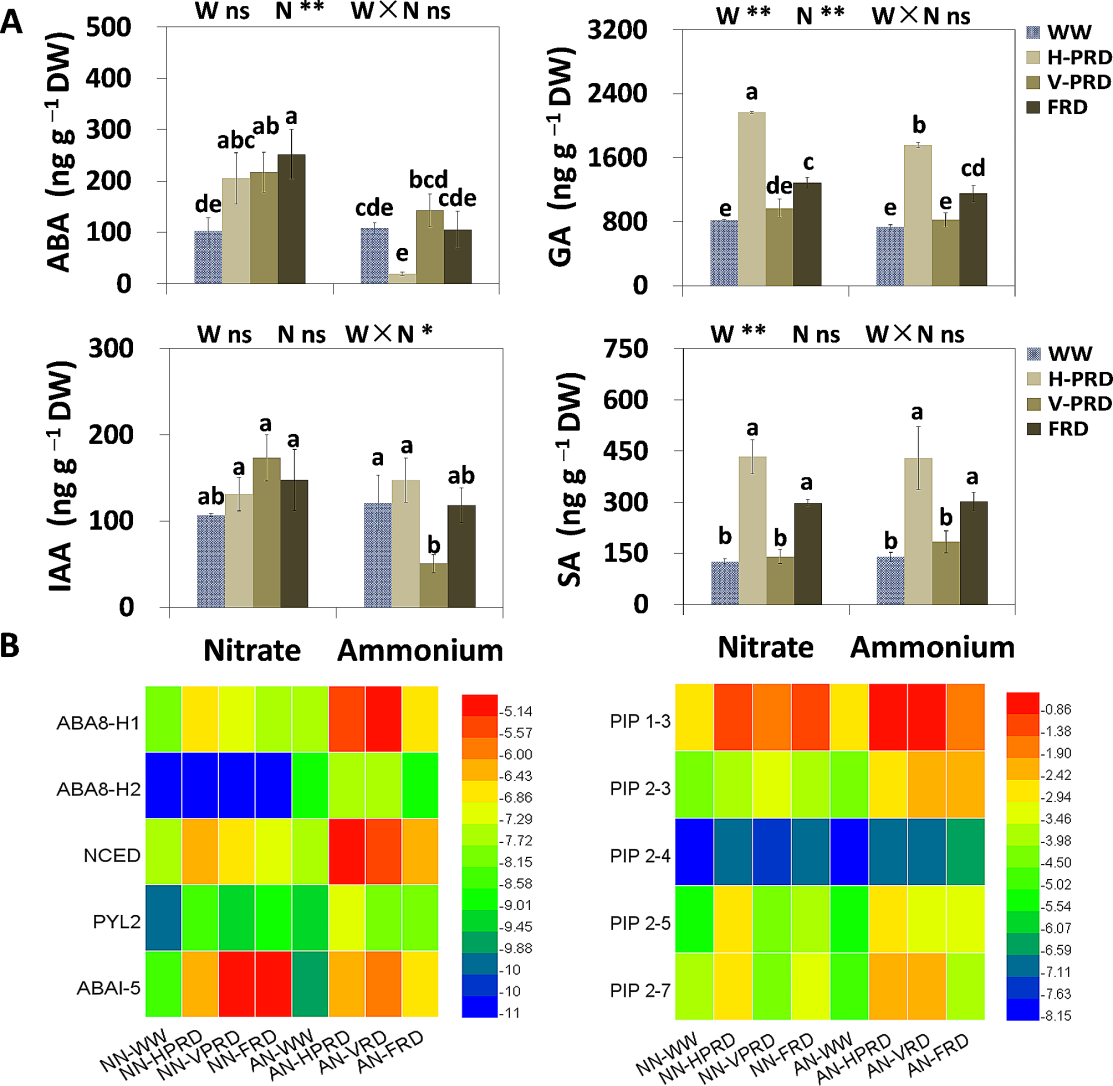
Analysis of drought-induced related phytohormone and expression levels of critical genes response to drought physiology. (**A**) Concentration of phytohormone (ABA, GA, IAA and SA) in leaves. (**B**) Heat map of transcriptional fold changes of genes involved in ABA signal pathway and *PIPs* relative to reference gene. The four water conditions (well-watered condition (WW), partial-root zone drought in horizontal (H-PRD) and vertical (V-PRD) direction, and full root-zone drought (FRD)) are combined with nitrate (NN) or ammonium (AN) treatment. Means ± SE are indicated by the columns and the bars above columns (*n* = 4). Statistically different values (*P* < 0.05) based on the multiple comparisons (Fisher’s LSD test) in ANOVA are indicated by different letters above the bars. The results of two-way ANOVA (water effect, nitrogen effect, and interaction effect) are listed as: *P < 0.05; **P < 0.01; ns, not significant

The concentration of ABA was increased by PRD and FRD when nitrate N was applied (Fig. 3A). Under V-PRD and FRD, ABA concentration was higher (52% and 138%, respectively) in nitrate treatment as compared with ammonium (Fig. 3A). SA and GA concentration were increased by H-PRD and FRD (Fig. 3A), Nitrate increased GA_3_ level under H-PRD while it increased IAA under V-PRD (Fig. 3A).

The transcription level of *NCED*, *ABA8-H1*, *ABA8-H2 and PYL2* all were higher in nitrate than that in ammonium treatment, while *ABI-5* showed the opposite trend. The transcript abundance of *NCED*, *ABI-5*, *ABA8-H1 and PYL2* all were up-regulated by PRDs and FRD in both N treatments (Fig. 3B). The transcription level of five *PIP* genes increased under H-PRD and FRD regardless of N forms, while these *PIP* genes elevated under V-PRD in ammonium treatment. The expression of *PIP1-3* and *PIP2-3* increased under V-PRD in nitrate treatment. Under H-PRD treatment, the relative expression of *PIP2-3* and *PIP2-7* were higher in ammonium compared to nitrate treatment. Under V-PRD, the relative expression of the five *PIP* genes were higher (52%, 13%, 7%, 23%, and 51%, respectively) in ammonium compared to nitrate. Under FRD treatment, the expression of *PIP2-3* and *PIP2-5* were higher (48% and 33%, respectively) in ammonium compared to control, whereas the expression abundance of *PIP1-3* was increased by 51% in nitrate treatment compared to control (Fig. 3B).

### Stem anatomical responses to partial root-zone drought as effected by N forms

**Fig. 4:**
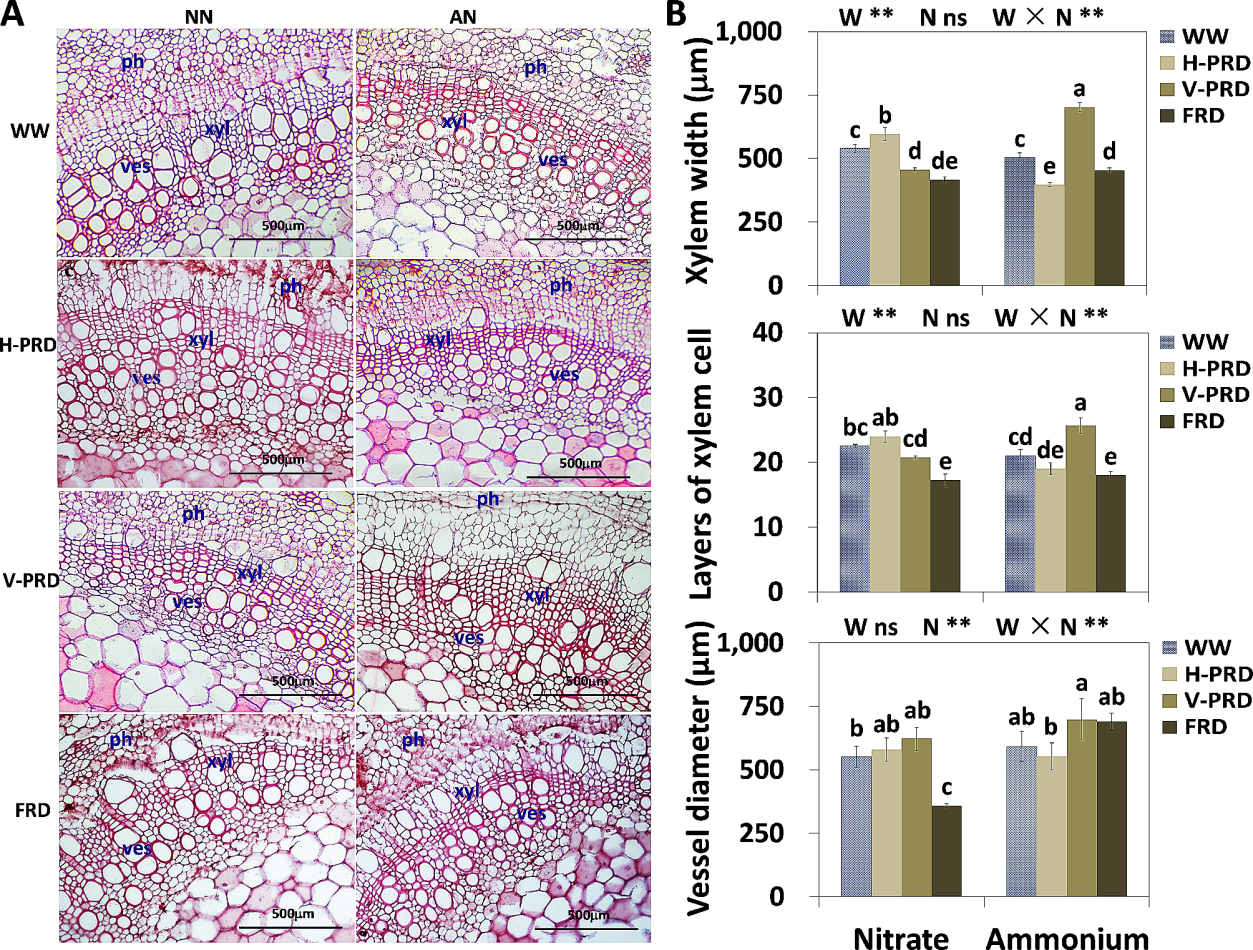
Stem anatomical properties of *Catalpa bungei* seedlings under varying water and nitrogen supply. (**A**) Anatomical diagram of the xylem. (**B**) Statistical analysis of xylem parameters. ph, phloem; xyl, xylem; ves, vessel. The four water conditions (well-watered condition (WW), partial-root zone drought in horizontal (H-PRD) and vertical (V-PRD) direction, and full root zone drought (FRD)) are combined with nitrate (NN) or ammonium (AN) treatment. Means ± SE are indicated by the columns and the bars above columns (*n* = 4). Statistically different values (*P* < 0.05) based on the multiple comparisons (Fisher’s LSD test) in ANOVA are indicated by different letters above the bars. The results of two-way ANOVA (water effect, nitrogen effect, and interaction effect) are listed as: *P < 0.05; **P < 0.01; ns, not significant

FRD inhibited xylem development by reducing xylem width and layers of xylem cell under both N conditions (Fig. 4). In nitrate treatment, xylem width was increased by 30% in H-PRD to that in V-PRD (Fig. 4). In contrast, xylem development was superior in V-PRD when NH_4_^+^ was applied, as indicated by greater xylem width and xylem cell layers. Under H-PRD, xylem development in nitrate was superior to that in ammonium treatment, while xylem in ammonium was superior to nitrate under V-PRD and FRD.

### PCA and Pearson’s correlation analysis

**Fig. 5:**
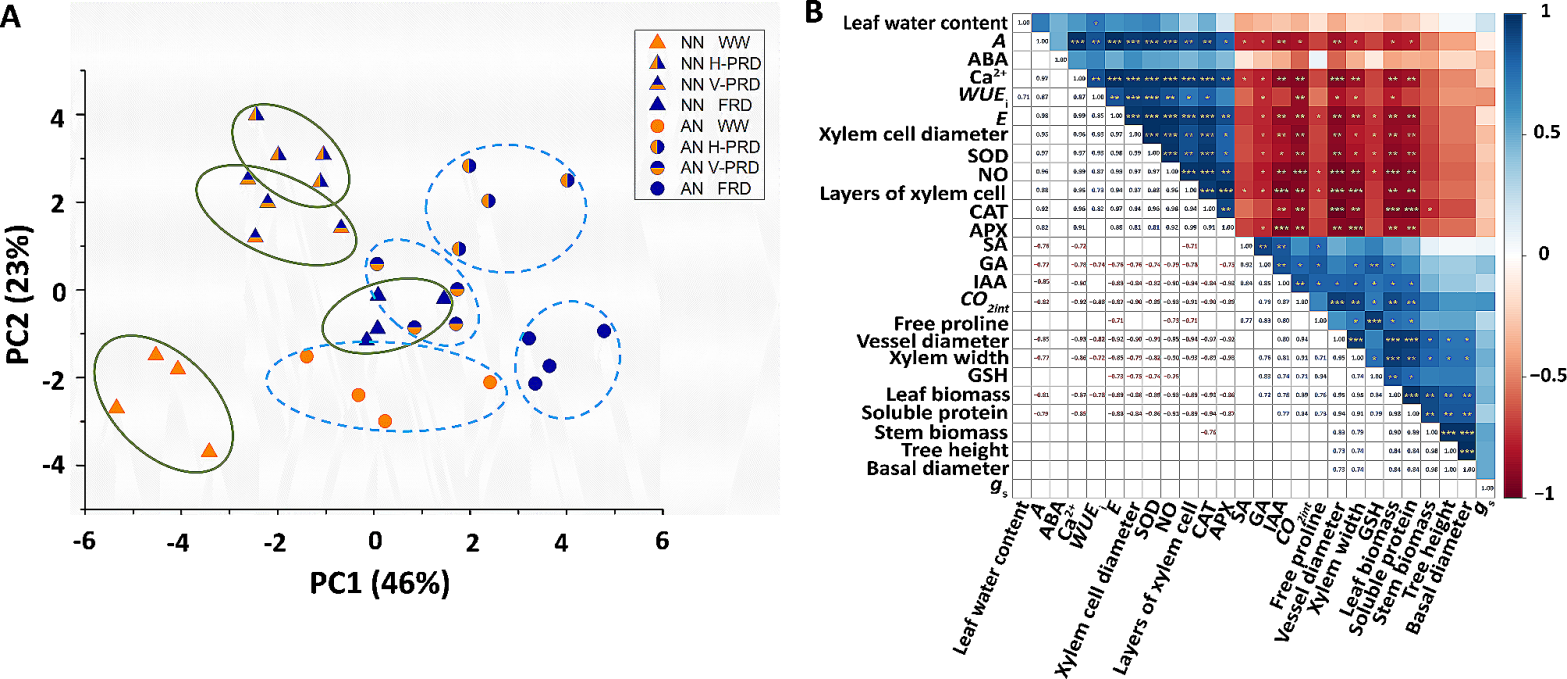
Correlation between growth, anatomical and physiological parameters under combined conditions of water and N forms. (**A**) Principal component analysis (PCA). (**B**) Pearson’s correlation analysis. The four water conditions (well-watered condition (WW), partial-root zone drought in horizontal (H-PRD) and vertical (V-PRD) direction, and full root zone drought (FRD)) are combined with nitrate (NN) or ammonium (AN) treatment. Traits related to physiological responses, seedling growth and xylem development are included in PCA and Pearson’s correlation analysis. *P < 0.05; **P < 0.01; ***P < 0.001

Physiological traits, seedling growth and xylem development were investigated by principal component analysis (PCA) (Fig. 5A). PC1 and PC2 accounted for 46% and 23% of the total variables, respectively. Water effect was uncoupled by both PC1 and PC2. Tree height, biomass of stem and leaf, basal diameter, soluble protein and Ca^2+^ were main components with positive scores in PC1 (Fig. 5A). The activity of antioxidants such as APX and CAT were main contributors of PC2.

Pearson’s correlation analysis showed that *WUE*_i_ was positively correlated with *E*, SOD, CAT and xylem cell layers, while *WUE*_i_ was negatively correlated with GA, xylem width and leaf biomass (Fig. 5B). Xylem width was positively correlated with the level of growth-promoting phytohormones such as IAA and GA, the concentration of free proline and the level of soluble protein and GSH. Leaf biomass was positively correlated with tree height, basal diameter and soluble protein (Fig. 5B).

## Discussion

### Effects of partial root-zone drought on growth and gas exchange parameters

Major growth traits and biomass accumulation were significantly inhibited under FRD regardless of nitrogen forms, while growth was less inhibited by PRDs than that by FRD treatment, as plants can maintain water uptake in the moist root-zone of PRDs so as to meet the water requirement for fundamental growth [13, 28]. It was reported that NH_4_^+^ may either enhance or inhibit plan growth, which depending on the other factors such as N concentration, soil pH, light condition, as well as the plant species being investigated [36, 37]. In the present study, major growth traits were superior in nitrate to that in ammonium treatment under both WW and PRDs conditions. However, major growth traits had no differences between the two N forms under FRD condition. This may be explained as the effect of N forms on seedling growth of *C. bungei* was covered up or interfered by the severe inhibitive effect of FRD.

To cope with drought stress, trees usually reduce stomata aperture and decrease transpiration rate to avoid excess water losses. During this process, leaf net photosynthetic rate can be limited due to the sharp decrement of *g*_*s*_ and intercellular *CO*_2int_ [38]. The present results showed that both of *A* and *E* declined upon FRD regardless of N form, mainly due to the sharp decrement of stomatal conductance. Interestingly, *WUE*_i_ was superior under FRD as compared with WW due to the active stomatal regulation trigged by ABA signals [8]. *PIPs* are responsible for the flow of water across the plasma membrane and promote leaf mesophyll conductance of CO_2_, and thereby enhance photosynthesis efficiency [39]. In the present study, most of the *PIPs* gene family members exhibited drought-induced expression under FRD. These results demonstrate that *C. bungei* cope with severe drought (FRD) by sharply decreasing stomata conductance, promoting *WUE*_i_ and improving water status by inducing the transcription of *PIPs* gene family.

In contrast to the sharp decrement of stomata conductance and gas exchange parameters (*A*, *E* and *g*_*s*_) under FRD, these parameters were less inhibited by PRD, mainly due to the lower extent of water deficit under PRD. Interestingly, *WUE*_i_ was significantly superior in V-PRD to H-PRD treatment, resulting from a markedly lower *E* in V-PRD treatment. Further analysis reveal that the level of ABA signal was higher under V-PRD. Moreover, transcriptional regulation of critical genes participating in the abscisic acid signaling pathway, *ABI-5* [40] was more active in V-PRD than that in H-PRD. These results indicate that the active ABA signaling should be responsible for the precise stomata regulation and superior *WUE*_i_ under V-PRD. This pattern may be related to the evolutionary adaptation of *C. bungei* in North China where periodical drought is common and V-PRD occurs frequently as surface soils usually dry faster than deeper soils.

### The influence of N forms on antioxidant defense and *WUE*_i_

Plants usually initiate a series of antioxidant defense and osmoregulatory processes to mitigate stress damage to the organism [8, 28]. In sugarcane plants supplied with NO_3_^−^, ROS accumulation under drought was lower than ammonium due to the enhanced activity of CAT, SOD and APX [41]. Under both PRDs and FRD in the present study, nitrate is superior to ammonium in terms of antioxidant defense, mainly due to the positive effect of nitrate on the production of antioxidants and osmoregulatory substances such as free proline, APX, GSH and soluble protein. This result provides important guidelines for water and fertilizer management in *C. bungei* plantations, as nitrate fertilization is preferred than ammonium for improving plant growth and stress resistance in drought prone areas.

Significant effect of N forms on stomata regulation and leaf *WUE*_i_ was detected, and stomata regulation seems more active and precise in nitrate than that in ammonium treatment. Interestingly, the mechanism underlying the superior *WUE*_i_ in nitrate treatment seems to be different between different water conditions. Under V-PRD condition, the superior *WUE*_i_ in nitrate treatment is mainly attributed to the active stomata regulation as indicated by lower *E* and *g*_s_. It is reported that ABA-dependent guard cell NO signaling elevate the level of intracellular Ca^2+^ ions and this signaling network activate SLAC1 and SLAH3 anion channels [42–44]. In the present study, nitrate promoted the activation of signaling network consisting of NO, Ca^2+^ and ABA, which contributed to the active stomata regulation and superior *WUE*_i_ in nitrate treatment. Moreover, ABA and SA were synchronously induced and they interactively participated in stomata regulation under H-PRD [28]. The superiority of improving water use efficiency through regulating stomata can enhance the adaptability of *C. bungei* to drought and alleviate the impact of drought on growth. Under FRD condition, the superior *WUE*_i_ in nitrate treatment mainly resulted from the superior *A*. As compared with ammonium, nitrate produces more active NO signal under water deficit which alleviate oxidative damage and benefit photosynthesis efficiency under drought stress [41]. Under FRD in the present study, nitrate enhanced the production of NO, which promoted antioxidant defense via elevating the level of free proline, GSH and soluble protein and thus benefited photosynthetic efficiency. Higher photosynthetic rate can produce more carbohydrates, which can maximize the yield of *C. bungei* in drought environment.

### The interactive effect of water and nitrogen forms on xylem development

Previous study showed that *WUE*_i_ was tightly correlated with carbohydrate production and xylem development [6, 8], and N application promoted xylem development of *C. bungei* under V-PRD condition via promoting the efficiency of carbohydrate production and enhancing C-N interaction [28]. In the present study, leaf biomass and xylem width both are positively correlated with soluble protein level, indicating that in addition to carbohydrate, soluble protein also plays a major role in maintaining plant growth and xylem development of *C. bungei* under PRDs. Moreover, *WUE*_i_ is positively correlated with SOD and CAT, indicating the critical roles of antioxidant enzymes in improving *WUE*_i_ and xylem development under drought.

Interestingly, the interactive effect of water and N forms was significant on wood xylem development of *C. bungei*. Under V-PRD, ammonium is more beneficial for wood development than nitrate. In contrast, nitrate rather than ammonium is preferred for wood development in H-PRD treatment. It was reported that some trees prefer to ammonium but not nitrate [45], mainly because the transformation of NO_3_^−^ to NH_4_^+^ is an energy-consuming process which requires two steps of reduction reaction [46]. However, ammonium may lead to toxicity due to the damages on cell membranes and cell walls by excessive accumulated extracellular H^+^ [47]. The present results indicate that the toxicity effect of ammonium may be significant under H-PRD and thus the xylem development is inhibited, while this toxicity effect may be less significant under V-PRD. The underlying mechanism of this interesting pattern deserves to be further addressed in the following researches.

## Conclusions

**Fig. 6:**
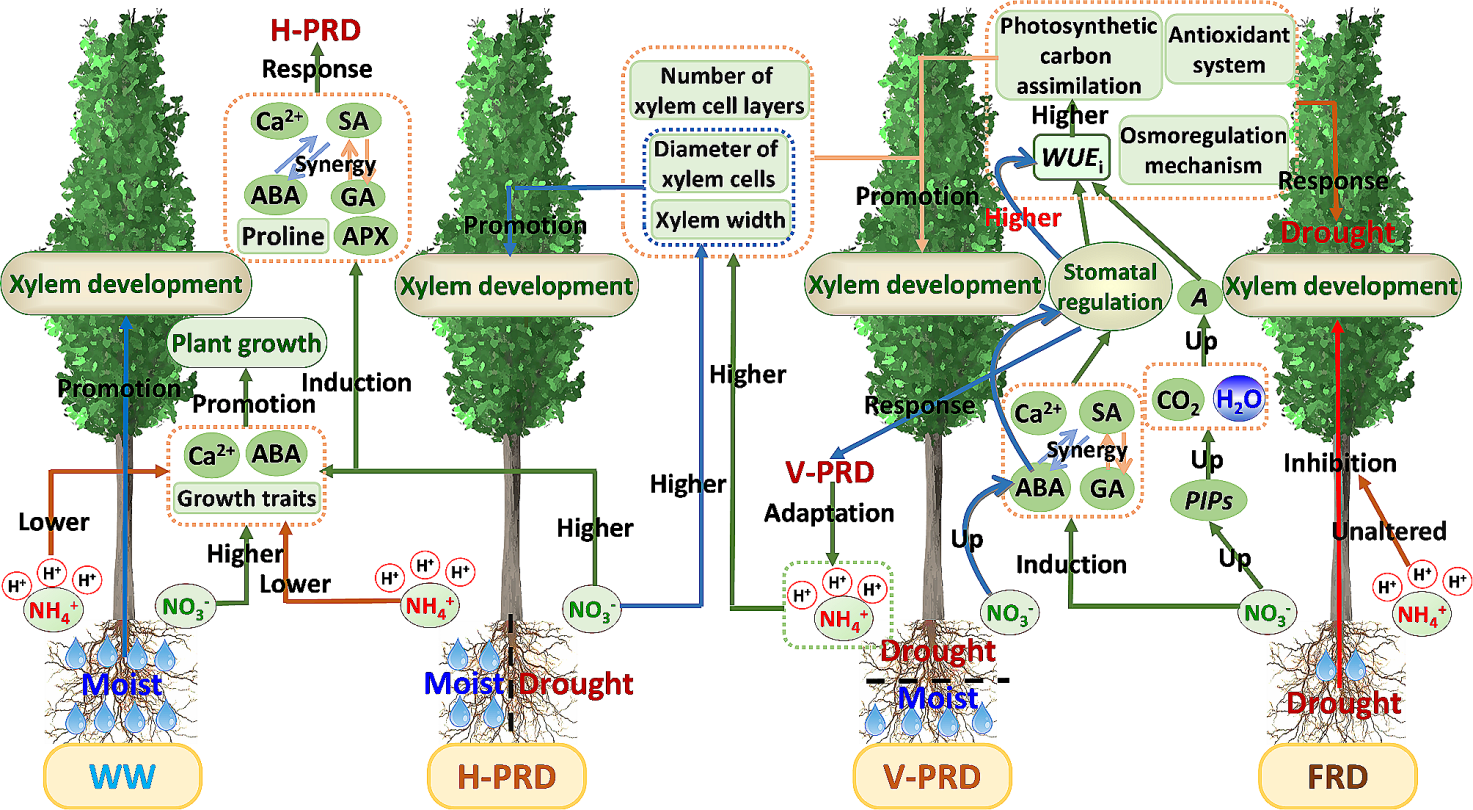
The schematic model of drought responses and xylem development of *Catalpa bungei* under diverse drought types as affected by nitrogen forms. *C. bungei* respond to severe drought (FRD) by sharply decreasing stomata conductance and increasing *WUE*_i_. Under FRD condition, the effect of N forms is covered up by the drastic drought effect. As compared with FRD, stomata conductance and gas exchanges were less inhibited by PRDs. Stomata regulation and *WUE*_i_ was superior in V-PRD to H-PRD due to active induction of ABA signaling. Under both PRDs and FRD, nitrate is superior to ammonium in terms of antioxidant defense, stomata regulation and leaf *WUE*_i_. Under V-PRD, nitrate benefits *WUE*_i_ via activating the signaling network of NO, Ca^2+^ and ABA which promotes stomata regulation. Under FRD, nitrate favors *WUE*_i_ via enhancing NO signal which promotes antioxidant defense and elevates photosynthetic efficiency. Soluble protein plays a major role in maintaining xylem development under PRDs, while the activity of SOD and CAT play critical roles in improving *WUE*_i_. The toxicity effect of ammonium on xylem development is significant under H-PRD while it is less significant under V-PRD condition

The physiological and anatomical characteristics of *Catalpa bungei* in partial and full root-zone drought under the influence of N forms were explored (Fig. 6). *C. bungei* respond to severe drought (FRD) by sharply decreasing stomata conductance and increasing *WUE*_i_. The effect of N forms under FRD condition is covered up by the drastic drought effect. As compared with FRD, stomata conductance and gas exchanges were less inhibited by PRDs. Stomata regulation and *WUE*_i_ was superior in V-PRD to H-PRD due to active induction of ABA signaling. Under both PRDs and FRD, nitrate is superior to ammonium in promoting drought tolerance and photosynthetic efficiency of *Catalpa bungei*. Nitrate play the key role via activating the signaling network of NO, Ca^2+^ and ABA, which facilitates stomata regulation, antioxidant defense and efficient photosynthesis. Interactive effect of water and N forms on xylem development was observed, and ammonium and nitrate is preferred for wood development in V-PRD and H-PRD treatment, respectively.

### Electronic supplementary material

Below is the link to the electronic supplementary material.

Supplementary Material 1: Figure S1: The schematic drawing of experimental design.

## Data Availability

Data is provided within the manuscript or supplementary information files.

## Declarations

### Experimental research and field studies on plants

All relevant institutional, national and international guidelines and legislation were compiled or adhered to in the production of this study.
