# Supplementary material for: Effects of nitrate- and ammonium- nitrogen on anatomical and physiological responses of Catalpa bungei under full and partial root-zone drought
Source: BMC Plant Biol. 2024 Mar 26;24:217. doi: 10.1186/s12870-024-04874-3 (PMC10964640; doi:10.1186/s12870-024-04874-3)
Supplement: Supplementary file 1 — Supplementary Material 1: Figure S1: The schematic drawing of experimental design. [file 12870_2024_4874_MOESM1_ESM.docx]

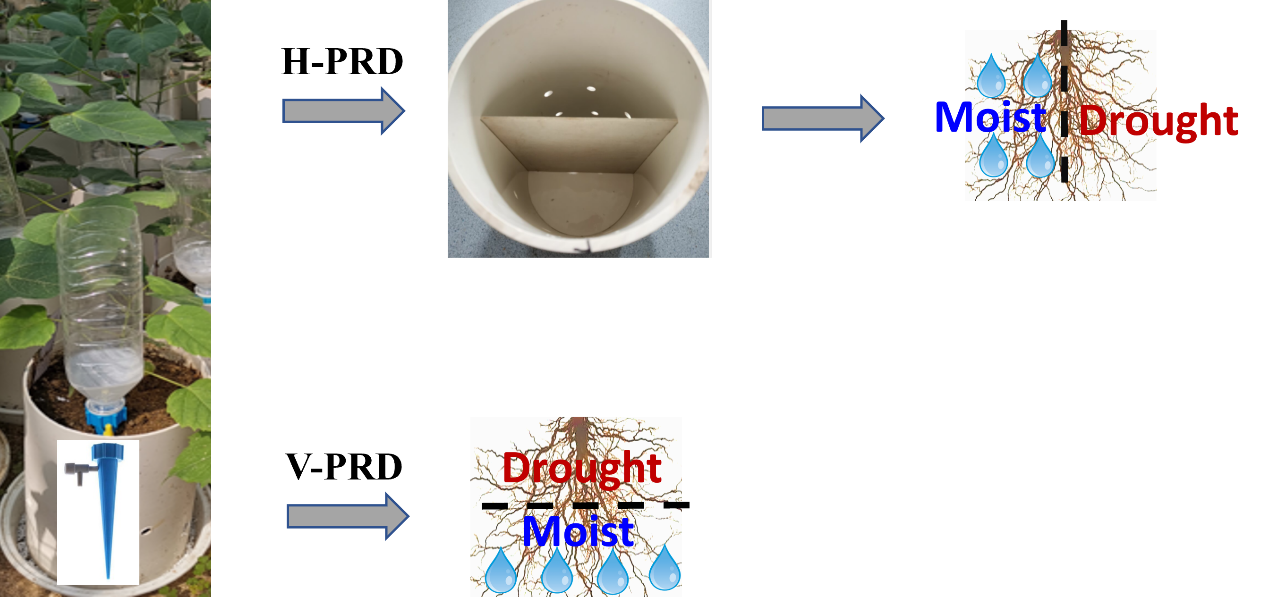

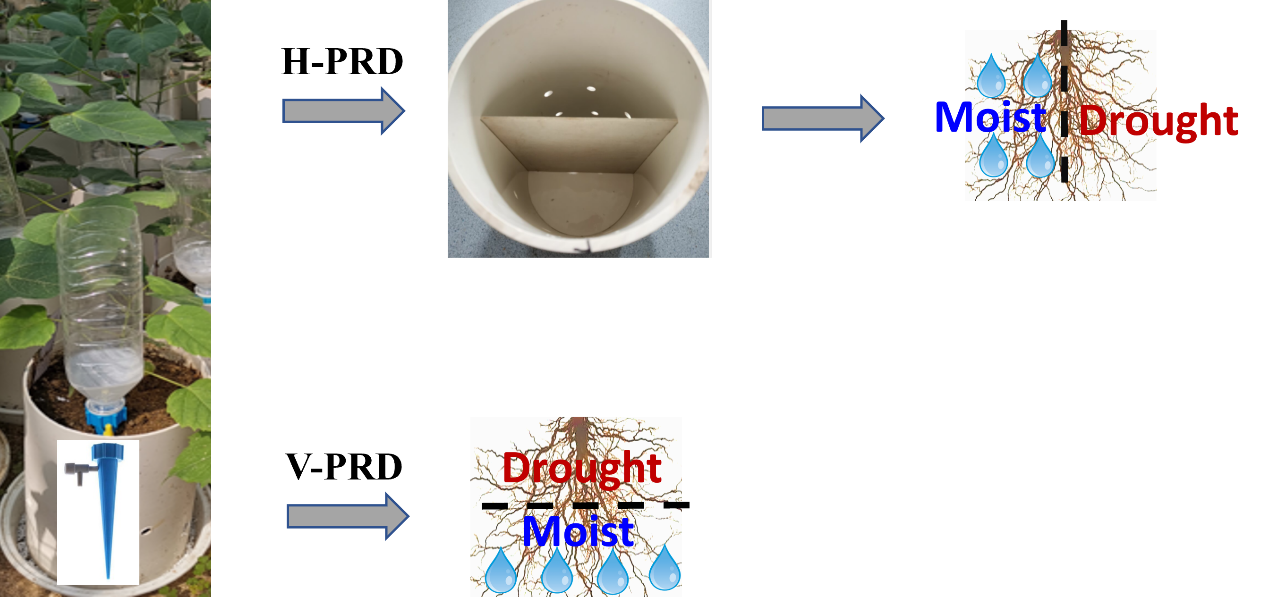

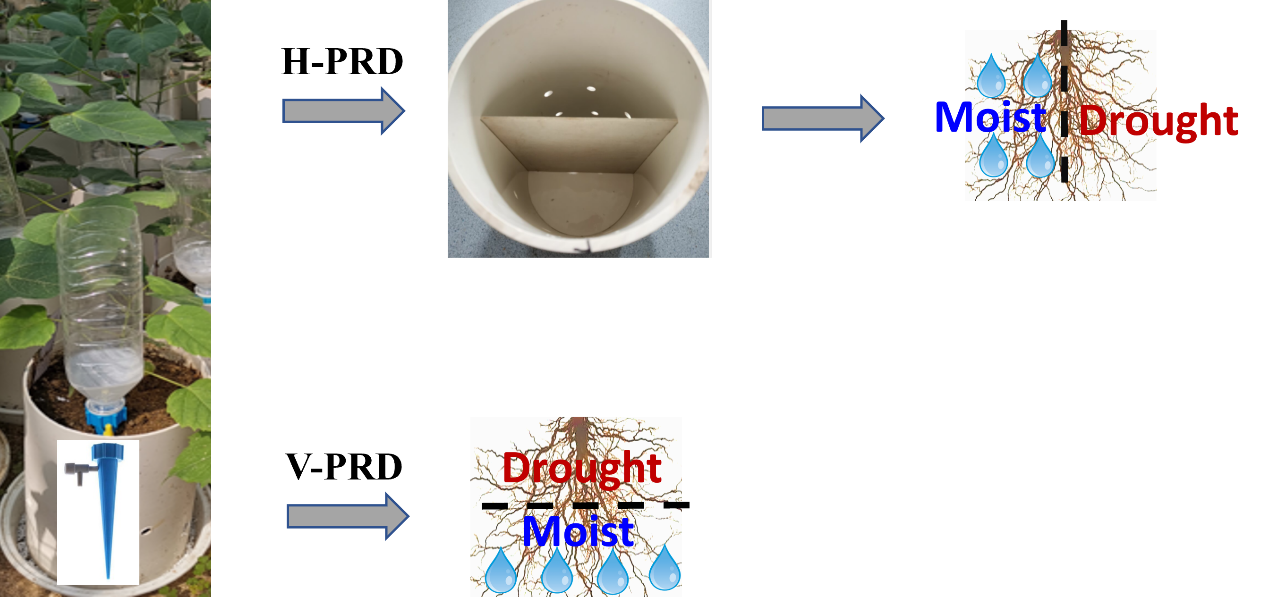

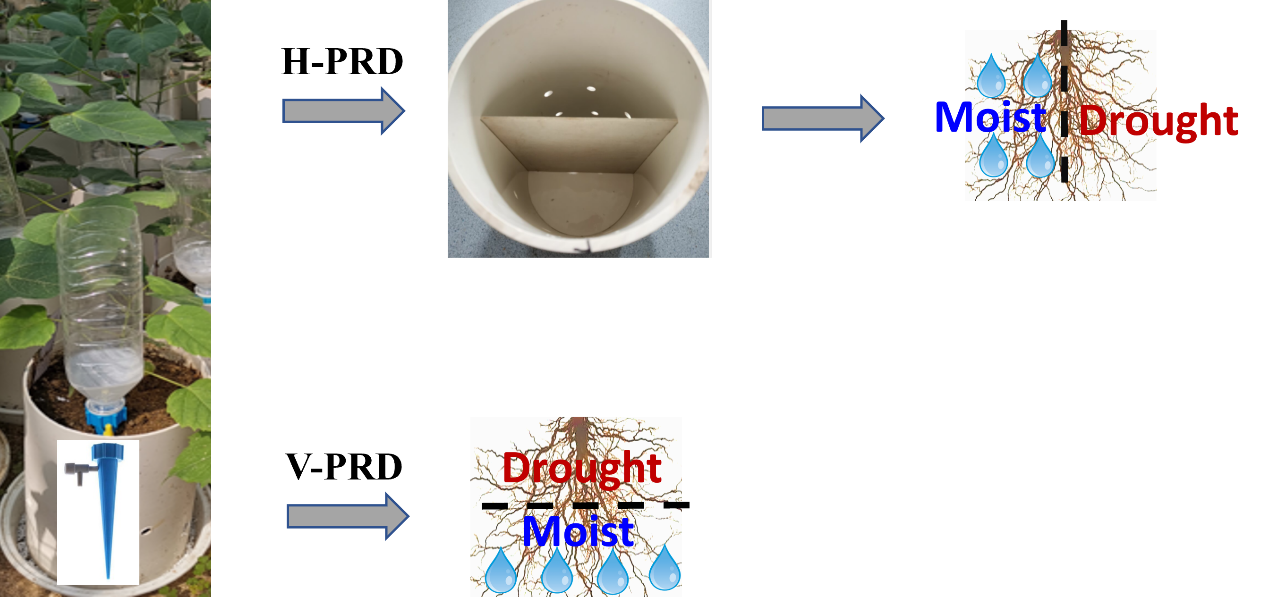


**Fig. S1 The schematic model of experimental design**

The soil moisture content of H-PRD treatment was 45±5% of field capacity on drought root zone and 70±5% of field capacity on moist root zone, respectively. The soil moisture content of V-PRD treatment was 45±5% of field capacity on the top soil region and 70±5% of field capacity on the bottom soil region. To achieve the soil moisture described above, a split-root pot framework was used in which each pot was divided into two compartments with plastic board, and drip irrigation device with water dropper in each compartment was applied. One water dropper was set in the dry zone and moist zone of the H-PRD treatment, respectively. Subsequently, one water dropper was set in the top and bottom zone of the V-PRD treatment, respectively. The time domain reflectometry apparatus (TDR300) was applied to detect soil water contents in different soil zones every day. The water flow in each water dropper was set to a slow rate to avoid water diffusion between different root zones. The total water supply for the V-PRD treatment and the H-PRD treatment was the same, which is half of WW treatment.

**V-PRD**

**H-PRD**
